# Dermatological Health in the Light of Skin Microbiome Evolution

**DOI:** 10.1111/jocd.16557

**Published:** 2024-09-09

**Authors:** Diala Haykal, Hugues Cartier, Brigitte Dréno

**Affiliations:** ^1^ Centre Laser Palaiseau Palaiseau France; ^2^ Centre Médical Saint Jean Arras France; ^3^ Department of Dermato‐Cancerology CHU Nantes—Hôtel‐Dieu CRCINA Nantes France

**Keywords:** bacteriotherapy, dysbiosis, microorganisms, phages, probiotic, skin aging, skin conditions, skin microbiome, skin microbiota, skin transplantation

## Abstract

**Background:**

The complex ecosystem of the skin microbiome is essential for skin health by acting as a primary defense against infections, regulating immune responses, and maintaining barrier integrity. This literature review aims to consolidate existing information on the skin microbiome, focusing on its composition, functionality, importance, and its impact on skin aging.

**Methods:**

An exhaustive exploration of scholarly literature was performed utilizing electronic databases including PubMed, Google Scholar, and ResearchGate, focusing on studies published between 2011 and 2024. Keywords included “skin microbiome,” “skin microbiota,” and “aging skin.” Studies involving human subjects that focused on the skin microbiome's relationship with skin health were included. Out of 100 initially identified studies, 70 met the inclusion criteria and were reviewed.

**Results:**

Studies showed that aging is associated with a reduction in the variety of microorganisms of the skin microbiome, leading to an increased susceptibility to skin conditions. Consequently, this underlines the interest in bacteriotherapy, mainly topical probiotics, to reinforce the skin microbiome in older adults, suggesting improvements in skin health and a reduction in age‐related skin conditions. Further exploration is needed into the microbiome's role in skin health and the development of innovative, microbe‐based skincare products. Biotherapeutic approaches, including the use of phages, endolysins, probiotics, prebiotics, postbiotics, and microbiome transplantation, can restore balance and enhance skin health. This article also addresses regulatory standards in the EU and the USA that ensure the safety and effectiveness of microbial skincare products.

**Conclusion:**

This review underscores the need to advance research on the skin microbiome's role in cosmetic enhancements and tailored skincare solutions, highlighting a great interest in leveraging microbial communities for dermatological benefits.

## 1 Introduction

Human skin, as big as 2 m^2^, is the largest organ. The skin microbiome, consisting of a wide range of microorganisms, has a crucial function in preserving skin health and balance. The skin microbiome has been significantly transformed by recent progress in sequencing technologies, leading to a profound enhancement in our comprehension of its composition, diversity, and function [1, 2]. The human skin microbiome is a diverse and ever‐changing ecosystem made up of a variety of microorganisms that live in different areas of the skin [2]. It is essential for skin health as it supports the skin's barrier function and immune system. The microbiome is formed in infancy and is shaped by genetics, environmental influences, and lifestyle decisions. Imbalance in the skin microbiota, known as dysbiosis, can result in a range of skin conditions such as infections, allergies, and autoimmune diseases [3]. Comprehending the structure and behavior of the skin microbiome is crucial for creating methods to enhance skin well‐being and address aging skin [4, 5]. Current research seeks to clarify the complex connection between the skin microbiome and host physiology, leading to new approaches in skincare and dermatology. The objective of this literature review is to consolidate existing information on the skin microbiome, with a specific emphasis on its composition, functionality, importance, and impact on skin aging. This piece addresses the evolution of skin microbiome from early life until old age, casting light on the most influential factors in its changes. Aging skin microbiome undergoes several types of changes depending on external and internal factors such as physiological age, lifestyle, diseases, exposome, etc. These changes verify the results of different therapeutic strategies. These therapies often go through some regulatory principles which require further improvement.

## 2 Methods

**FIGURE 1:**
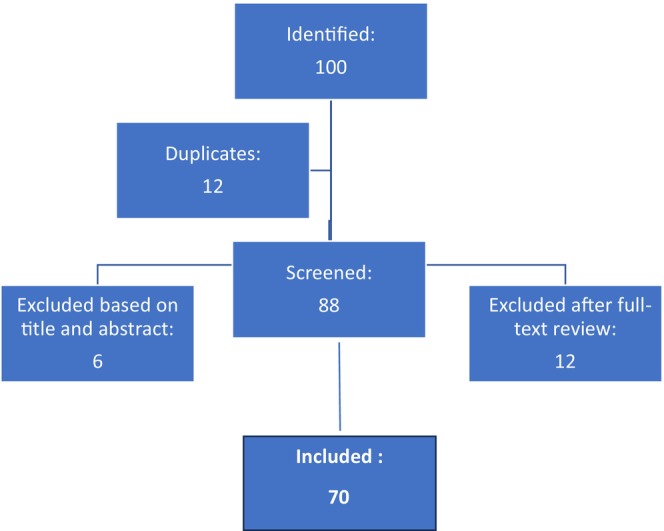
Flowchart.

An exhaustive exploration of scholarly literature was performed utilizing electronic databases including PubMed, Google Scholar, and ResearchGate. Relevant studies published between 2011 and 2024 were identified using keywords: “skin microbiome,” “skin microbiota,” and “aging skin.” Studies were included if they involved human subjects, focused on the skin microbiome and its relationship with skin health, and provided clear definitions and measurements of dermatological health. A total of 100 studies were initially identified through this search process. Out of the 100 identified studies, 12 duplicates were excluded, resulting in 88 studies that were screened based on their titles and abstracts. During this screening process, an additional six studies were excluded due to irrelevance with preliminary inclusion criteria. Subsequently, 82 studies were assessed for full‐text eligibility. Upon further review, 12 studies were excluded owing to divergence of their content from the main topic of the present study. Finally, 70 studies met the criteria for relevance with skin microbiome and skin wellness and were thus considered eligible for inclusion in the review (Figure 1).

The included studies were categorized based on a holistic approach to the evolution of the skin microbiome. With this perspective in mind, the present review is composed of five sections, covering various aspects of skin microbiome and its impact on overall skin health, in addition to regulatory frameworks and future perspectives (Table 1).

**TABLE 1:** Summary.

References	Authors	Year	Findings	Type of research
1	Eisenstein	2020	Describes the complex ecosystem of the skin microbiome and its importance for skin health	Review
2	Grice et al.	2011	Explores the skin microbiome and its interactions with the human host	Review
3	Skowron et al.	2021	Examines the impact of intrinsic and extrinsic factors on skin microbiota composition	Review
4	Smythe et al.	2023	Reviews research on skin microbiome and its effects on aging	Review
5	Harris‐Tryon et al.	2022	Discusses the role of the microbiota in maintaining skin barrier function	Review
6	Habeebuddin et al.	2022	Investigates the potential benefits of topical probiotics for balanced skin microbiota	Review
7	Serghiou et al.	2023	Provides insights into recent research on early life skin microbiome	Review
8	Scharschmidt et al.	2013	Explores the ecology, genomics, and therapeutic potential of the skin microbiome	Review
9	Xu et al.	2019	Analyzes the relationship between acne, the skin microbiome, and antibiotic treatment	Review
10	Moitinho‐Silva et al.	2022	Studies the association between host genetic factors and skin microbiota composition	Observational study
11	Boxberger et al.	2021	Highlights the role of intrinsic and extrinsic factors in shaping the bacterial communities of skin microbiome	Review
12	Yang et al.	2022	Summarizes recent advances in digital microfluidic‐based platforms in evaluating skin microbiome	Review
13	Gallo et al.	2017	Discusses the role of the skin as an epithelial surface for microbial interaction	Commentary
14	Callewaert et al.	2020	Examines how changes in lifestyle and living environment can impact the skin microbiome and skin health and disease in humans	Review
15	Zhu et al.	2023	Analyzes the balance between health and disease in human skin bacterial microbiota	Review
16	McCarthy et al.	2022	Investigates the altered skin and gut microbiome in hidradenitis suppurativa	Observational study
17	Chambers et al.	2020	Reviews the impact of aging on skin barrier immunity	Review
18	Baldwin et al.	2017	Discusses the importance of maintaining cutaneous microbiota harmony for skin health	Review
19	Johnson et al.	2018	Explores new molecular targets to promote wound healing through the cutaneous microbiome	Review
20	Demessant‐Flavigny et al.	2023	Reviews the role of *Staphylococcus aureus* in atopic dermatitis and skin microbiome dysbiosis	Review
21	Dessinioti et al.	2024	Analyzes perspectives for treating acne through the microbiome	Review
22	Myers et al.	2023	Identifies potential microbial features associated with skin aging	Multi‐study analysis
23	Ratanapokasatit et al.	2022	Analyzes the skin interactome role in skin aging and skin health	Review
24	Nakatsuji et al.	2021	Explores mechanisms for controlling skin immune function through the microbiome	Review
25	Hurlow et al.	2011	Reviews the issue of dry skin in older adults	Review
26	Jugé et al.	2018	Examines shifts in skin microbiota composition across aging in Western European women	Research study
27	Dréno et al.	2016	Updates dermatologists on the healthy skin microbiome	Review
28	Byrd et al.	2018	Provides a comprehensive overview of the human skin microbiome	Review
29	Kano et al.	2013	Shows that fermented milk containing *Bifidobacterium breve* benefits skin condition in healthy adult women	Clinical trial
30	Woo et al.	2024	Reviews the interaction between the microbiota and the skin barrier in aging skin	Review
31	Sharma et al.	2016	Explores anti‐aging effects of probiotics on the skin	Review
32	Dimarzio et al.	2008	Shows that topical application of bacterial sphingomyelinase increases skin‐ceramide levels in aged subjects	Clinical trial
33	Notay et al.	2020	Analyzes the cosmetic improvement of facial wrinkles using topical *Nitrosomonas eutropha*	Clinical trial
34	Weyrich et al.	2015	Examines associations between altered microbial communities and skin diseases	Review
35	Rocha et al.	2018	Explores the relationship between skin barrier function and the microbiome in acne	Review
36	Kang et al.	2009	Shows antimicrobial activity of enterocins against *Propionibacterium acnes*	Experimental study
37	Lee et al.	2019	Provides a comprehensive review of the potential role of the microbiome in acne	Review
38	Thio et al.	2018	Discusses the microbiome perspective in psoriasis and psoriatic arthritis	Review
39	França et al.	2021	Reviews the use of topical probiotics in dermatological therapy and skincare	Review
40	Guéniche et al.	2006	Shows improvement in atopic dermatitis symptoms by *Vitreoscilla filiformis* bacterial extract	Clinical trial
41	Guéniche et al.	2008	Demonstrates improvement in seborrheic dermatitis with *Vitreoscilla filiformis* biomass	Clinical trial
42	Chen et al.	2013	Discusses current perspectives and future challenges in skin microbiome research	Review
43	Carvalho et al.	2023	Reviews the impact of cosmetic products on the skin microbiota	Review
44	Bouslimani et al.	2019	Analyzes the dynamics of skin chemistry and microbiome with the use of skincare products	Observational Study
45	Wallen‐Russell et al.	2019	Explores the effects of everyday cosmetics on the skin microbiome using biodiversity as a measure	Observational study
46	Ch'ng	2024	Shows the effectiveness of an emollient “plus” in rebalancing the skin microbiome for managing atopic dermatitis.	Clinical trial
47	Sarasati et al.	2023	Investigates the potential of plant‐derived exosome‐like nanoparticles for biomedical and regenerative applications	Experimental study
48	Bai et al.	2024	Examines clinical applications of exosomes in cosmetic dermatology	Review
49	Ghasemian	2021	Explores the application of exosome‐derived mesenchymal stem cells in treating fungal diseases	Experimental study
50	Dagnelie et al.	2020	Highlights the role of bacterial extracellular vesicles in inflammatory dermatoses	Review
51	Hendricks et al.	2019	Discusses the knowns, unknowns, and emerging trends of skin bacterial transplants in atopic dermatitis	Review
52	Natarelli et al.	2023	Explores the role of bacteriophages and the phageome in dermatology	Review
53	Özal et al.	2022	Provides a systematic review of bacteriophages and endolysins for reducing microorganisms	Systematic review
54	Castillo‐González et al.	2022	Discusses the therapeutic potential of bacteriotherapy with human skin commensals in atopic dermatitis	Review
55	Dewi et al.	2023	Examines the possibilities of bacteriotherapy for skin aging and atopic dermatitis	Review
56	Smirnova et al.	2023	Reviews new bacteriotherapy approaches for inflammatory skin diseases	Review
57	Ito et al.	2022	Shows the effect of emollients containing vegetable‐derived lactobacillus in treating atopic dermatitis symptoms	Review
58	Park et al.	2014	Studies the transfer of skin microbiota between dissimilar autologous microenvironments	Clinical trial
59	Perin et al.	2019	Investigates the effects of human skin microbiota transplantation in models of atopic dermatitis	Pilot study
60	Myles et al.	2016	Discusses regulatory aspects of probiotics and microbial products in skincare	Experimental study
61	Wright et al.	2020	Reviews the role of regulatory agencies in pharmaceuticals and cosmetics	Review
62	Yingling et al.	2000	Explores the evolving use of probiotics and postbiotics in cosmetics and their regulatory aspects	Review
63	Arora et al.	2023	Analyzes the applications of machine learning and deep learning in microbiome research	Review
64	Hernández et al.	2022	Reviews challenges and best practices in applying machine learning to microbiome research	Review
65	Papoutsoglou et al.	2023	Highlights the potential of biosensors and AI in dermatology	Review
66	Haykal	2024	Introduces biosensors and their applications	Review
67	Bhalla et al.	2016	Explores the use of flexible and printable integrated biosensors for monitoring skin conditions	Review
68	Wang et al.	2023	Discusses dermal tattoo biosensors and their potential applications	Experimental study
69	Dhond et al.	2023	Reviews the impact of modern environments on skin microbiome, barrier integrity, and immune programming	Review
70	Prescott et al.	2017	Highlights the role of modern environments on skin microbiome and health	Review

## 3 Results

### 3.1 Skin Microbiome Composition

The skin microbiome consists mainly of bacteria, fungi, and viruses making up a wide range of microbial species. A single square centimeter contains up to one million microorganisms. The skin microbiome, consisting of fungi, viruses, archaea, and mites, starts forming at birth and experiences substantial transformations over human lifetime [6]. During birth, the skin is first inhabited by germs from the mother's microbiota and the nearby surroundings. Early colonization is affected by factors such as delivery method (vaginal birth or cesarean section), infant food (breastfed or formula‐fed), and antibiotic use. Infants delivered vaginally acquire their initial microbiota from their mothers' vaginal and fecal flora, fostering a microbial community that supports healthy immune and metabolic development. Conversely, cesarean‐delivered infants often have a microbiome more reflective of skin and hospital environments, which may predispose them to allergies, asthma, and obesity. The nature of an infant's diet also shapes their gut microbiome; breastfed infants generally harbor a microbiome rich in beneficial bacteria like *Lactobacillus* and *Bifidobacterium*, supported by human milk oligosaccharides that promote a protective gut environment. Formula‐fed infants, however, tend to develop a more varied microbiota from an earlier age, which can impact immune function differently by potentially reducing the abundance of beneficial bacteria such as *Bifidobacterium* and *Lactobacillus*, leading to an increased risk of allergies, asthma, and obesity due to less optimal development of the gut and skin immune systems [7, 8]. Additionally, antibiotic use during early life can disrupt these microbial communities, reducing diversity and depleting key bacterial populations which can have lasting effects on the child's health, increasing susceptibility to infections and possibly influencing lifelong health outcomes [9].

The skin microbiome changes as we get older due to genetic factors, environmental exposures, lifestyle choices, and physiological changes in the body as well. Genetic predispositions influence skin reactions and microbial resilience, while environmental factors like UV radiation and pollution can alter microbial habitats and introduce harmful species. Lifestyle choices, including diet and skincare routines, directly impact microbial balance; for example, high‐fat diets and harsh soaps can disrupt this balance, whereas nutrient‐rich foods and gentle skincare products support it [10]. Additionally, physiological changes such as decreased sebum production, thinner skin, and altered pH levels provide a different environment that affects microbial growth, potentially leading to skin conditions linked to aging. Hormonal changes throughout puberty can impact the skin's sebum production and microbial composition, resulting in disorders such as acne. During maturity, the microbiome reaches a stable state but remains susceptible to exposome and influences including food, hygiene habits, skincare products, and exposure to contaminants [11]. Consequently, imbalances in the skin microbiome have been associated with a range of skin disorders such as eczema, psoriasis, acne, and rosacea. It is essential to comprehend the evolution and alterations in the skin microbiome from infancy to old age to enhance skin health and address dermatological issues effectively [12].

### 3.2 Role of the Skin Microbiome in Dermatological Health

The common notion that human skin has an average surface area of 2 m^2^ fails to acknowledge the complexity of the skin, which includes approximately 5 million appendages, potentially elevating the total surface area to approximately 25 m^2^, making it the largest organ [13]. The 2–3 mm deep outer layer of the skin not only serves as an immunological barrier that protects the body from environmental factors, injuries, and infections, but also regulates temperature, prevents water loss, and facilitates vitamin D synthesis, highlighting the crucial role of the skin microbiome in maintaining overall skin health [2]. Skin health, in the field of dermatology within broad terms, refers to the optimal functioning and appearance of the skin, encompassing its protective barrier functions, temperature regulation, and support for overall bodily health. From a holistic approach, skin health is seen as the harmonious balance of all skin components, including the skin microbiome, immune system, and structural integrity, which work together to maintain the skin's resilience and its ability to protect against environmental stressors and infections. Accumulating evidence indicates that the skin microbiome has a vital function in preserving the balance of the skin and defending against harmful microorganisms. Commensal microorganisms engage in competition with potential pathogens for colonization sites and nutrients, thereby inhibiting the excessive growth of pathogenic species [14, 15]. Furthermore, the skin microbiome plays a role in controlling nearby immune reactions (innate immunity), impacting pathways related to inflammation and defense against microorganisms [3, 16]. The skin microbiome itself is integral to the modulation of these immune activities [17]. Commensal bacteria on the skin contribute to the education and regulation of the immune system including both innate and adaptative immunity, teaching immune cells to tolerate beneficial microbes while remaining vigilant against pathogens [18]. The imbalance of the skin microbiome has been linked to several dermatological conditions, such as acne vulgaris, atopic dermatitis (AD), psoriasis, wound infections, and skin aging [19, 20, 21, 22, 23].

### 3.3 Microbiome Diversity in Aging Skin: Impact on Aging Process and Dermatological Diseases Development

Research examining the relationship between aging skin and the skin microbiome has yielded insightful findings. This includes studying the impact of microbial metabolites, immune modulation, and tissue remodeling on age‐related changes in the skin [24]. To be specific, as the skin microenvironment changes by age, so does the skin microbial composition. To name a few of these changes, small blood vessels, sebaceous glands, and sweat glands shrink over time along with a decrease in nutrient supply [25]. In this regard, Jugé et al. [26] conducted a study revealing age‐related changes in microbial diversity, noting a decline with age and an increase in certain pathogenic species such as Proteobacteria and *Corynebacterium* spp. To investigate the impact of skin microbiome on different aging processes, Dréno et al. conducted a longitudinal investigation that explored the impact of chronological aging and photoaging on the skin microbiome, revealing distinct microbial signatures associated with different aging processes. They found that the skin microbiome changes significantly with age. This shift in microbiome composition is linked to decreased sebum production, thinner skin, and altered pH levels, creating a more favorable environment for pathogenic species and contributing to age‐related skin conditions. In younger individuals, there's a higher diversity of bacteria, including more *Cutibacterium* species, which thrive in oily skin. As skin ages, microbial diversity decreases, with an increase in species like *Staphylococcus* and *Corynebacterium*, linked to reduced sebaceous activity and immune changes. Photoaging further alters the microbiome, increasing species associated with dry, less resilient skin. These microbial shifts may contribute to the clinical signs of aging skin, such as dryness and reduced elasticity [27]. Furthermore, review articles by Byrd et al., Kano et al., and Woo et al. provided comprehensive overviews of the impact of the skin microbiome on age‐associated skin conditions and explored the effects of probiotic supplementation on the skin microbiome of elderly individuals, respectively [28, 29, 30]. Byrd, Belkaid, and Segre [28] refer to the diversity of microbial dermatological disease drivers in diabetic and obese elderly. Kano et al. [29] have referred to certain diets such as yogurt intake to have direct positive effect on adult females with chronic constipation and dry skin. Woo and Kim [30] point out that gut dysbiosis is directly involved in the skin microbiome and various dermatological conditions such as acne, eczema, rosacea, prurigo nodularis, and skin cancer. Collectively, these studies highlight the intricate interplay between aging skin and the skin microbiome, suggesting potential avenues for interventions to maintain skin health and mitigate age‐related skin changes. Other investigations conducted by Habeebuddin et al., Chambers et al., Sharma et al., Dimarzio et al., and Notay et al. shed light on the potential of topical probiotics to improve skin health and modify the skin microbiome among the elderly, thereby indicating the efficacy of therapeutic cosmetic treatments [6, 17, 31, 32, 33]. Their results suggest that a topical probiotic at a high concentration effectively reduces the severity of facial wrinkles in contrast to lower concentrations. Overall, these studies emphasize the dynamic relationship between aging skin and the skin microbiome, underscoring the importance of microbial dysbiosis in age‐related skin changes and highlighting avenues for further research and intervention development. Additionally, research by Weyrich et al. [34] focused on the role of the skin microbiome in age‐related inflammatory skin conditions, demonstrating dysbiosis and alterations in microbial metabolites in elderly individuals with dermatitis. Furthermore, several research investigated the direct relationship between skin microbiome dysbiosis and dermatological conditions such as rosacea, acne vulgaris, psoriasis, and AD [12, 35, 36, 37, 38, 39, 40, 41, 42]. Moreover, changes in the microbiome composition can influence systemic immune responses, potentially affecting overall health and susceptibility to other diseases. This compromised barrier function can lead to an increased susceptibility to skin infections and colonization by potentially pathogenic microorganisms. For instance, older adults are more prone to infections like cellulitis, fungal foot infections, and shingles, which are caused by the reactivation of the varicella‐zoster virus.

Overall, the skin interactome combines the genome, microbiome, and exposome, impacting skin aging and health [14]. We must now focus on the maintenance of ideal skin conditions through reducing the negative impacts of factors affecting the skin interactome to prevent premature aging. Although there is a lack of sufficient clinical research on skin anti‐aging treatments, the increasing interest in this developing field highlights the need for more investigation, which could lead to future therapeutic breakthroughs.

### 3.4 Microbiome‐Mediated Responses to Cosmetic Procedures

There is a critical need for more comprehensive, long‐term studies to investigate the impact of cosmetic skincare products and procedures, including laser therapy, chemical peels, and injectables, on the skin microbiome. However, emollients containing prebiotics have been shown to support the growth of beneficial skin bacteria, maintaining the skin barrier and microbial balance. Furthermore, understanding how changes in the microbiome affect treatment outcomes and post‐procedure complications is essential for grasping the intricate dynamics of microbiome alterations, highlighting a crucial area of focus in healthcare [43]. In addition, there is a need to create prediction algorithms that may customize therapies according to individual microbiome profiles to improve safety and effectiveness. There are several studies highlighting the effects of cosmetic procedures and products on the skin microbiome, revealing various outcomes based on the nature of the products used and the specifics of the procedures involved. One study explored the dynamics of the skin microbiome over a 9‐week period involving regular use of different personal care products like deodorants and moisturizers. This research demonstrated that these products alter the microbial diversity of the skin through the persistence of product ingredients like polyethylene glycol in the skin's microbiome [44]. Another research focused on the impact of everyday cosmetics on skin health, particularly how synthetic ingredients might damage the skin microbiome. This study used different types of face washes, including those with synthetic ingredients versus completely natural ones, to evaluate their effects on skin's microbial diversity, pH, moisture, and trans‐epidermal water loss. The findings suggested a potential link between synthetic cosmetics and reduced microbiome biodiversity, which might impair skin health and thereby accelerate aging [45]. Another study explores the critical role of the skin microbiome in managing AD, emphasizing that dysbiosis can exacerbate the condition by disrupting the skin barrier and immunity. It highlights the effectiveness of an emollient “plus,” which includes active ingredients such as *Vitreoscilla filiformis* and microresyl, designed to rebalance the skin microbiome and reduce inflammation. This type of emollient has been shown to modulate the skin microbiome more effectively than standard emollients, thereby improving AD symptoms and reducing disease severity [46]. Additionally, exosomes, small extracellular vesicles crucial for cell communication, have been researched for their potential impact on the skin microbiome, with studies suggesting that they significantly contribute to skin health. By facilitating communication between cells by transferring proteins, lipids, and genetic materials, exosomes can affect the behavior and composition of the skin microbiota. This interaction could help enhance skin barrier functions, modulate immune responses, and directly affect microbial composition, promoting a healthier skin environment. Both plant and human‐derived exosomes contribute uniquely to skin health through their interactions with the skin microbiome, each leveraging their molecular properties to support skin integrity and function. Specifically, plant‐derived exosomes provide immediate, surface‐level benefits such as enhanced protection and hydration, while human‐derived exosomes contribute to deeper, more systemic improvements in skin health and cellular communication [47, 48]. Although the precise mechanisms and outcomes of these interactions remain under active investigation, preliminary findings suggest that dermatological treatments utilizing exosomes hold promise for maintaining or restoring a balanced skin microbiome, thus opening new pathways for innovative therapeutic strategies [49, 50].

### 3.5 The Impact of Bacteriophages, Endolysins, Probiotics, and Skin Transplantation on the Microbiome

The current section will review the transformative potential of bacteriotherapy in dermatology, focusing on its efficacy in managing the skin microbiome and enhancing skin health amidst aging. Biotherapeutic approaches include using phages, endolysins, probiotics, prebiotics, postbiotics, and microbiome transplantation to restore balance and enhance skin health. Research has shown reassuring results in using targeted bacterial therapies to combat pathogenic bacteria and reinforce the skin's natural defenses [51, 52, 53].

Bacteriophages and endolysins are assessed for their targeted antimicrobial actions which could selectively eliminate pathogenic bacteria, thereby restoring a balanced skin microbial environment. The review by Ito and Amagai dives into the implications of skin microbiota for health and therapeutic strategies, emphasizing the reliability of bacteriotherapy to treat skin conditions. Bacteriotherapy involves using live bacteria or their products to modify the skin microbiome, aiming to restore balance and enhance skin health. This approach is particularly highlighted in the context of treating skin diseases such as AD, where specific beneficial bacteria or their enzymes are applied to the skin to improve barrier function and reduce pathogenic bacteria [54, 55, 56]. The therapeutic potential of these strategies is substantial, given their ability to harness the natural properties of the skin's microbiota. However, the review does not specifically focus on aging but rather on broader applications, suggesting that further research is needed to explore and optimize these bacterial therapies for various skin health aspects, potentially including aging in future studies [57].

The roles of pro‐, pre‐, and postbiotics are explored in terms of their ability to foster a beneficial microbial flora, enhance skin barrier functions, and potentially reverse the effects of aging. Probiotics introduce beneficial bacteria to the skin, improving its natural defenses and barrier integrity, while prebiotics nourish these beneficial bacteria, promoting a balanced microbiome that supports overall skin health [58]. Postbiotics offer additional benefits such as anti‐inflammatory and antioxidant properties, which are essential for repairing skin damage and reducing signs of aging. Together, these biotic elements create a synergistic approach to skincare, aiming to maintain skin resilience, hydration, and youthful appearance through advanced bioactive ingredients [33].

Another emerging scientific consensus points out that the skin microbiome plays a fundamental role in both the success of skin transplants and the management of skin diseases. Skin transplantation, especially in the context of burn injuries or chronic wounds, can significantly interact with and alter the skin microbiome. The transplanted skin not only brings its own microbiome but also needs to integrate with the recipient's existing microbial communities [59]. This integration is crucial for the success of the transplant, as a balanced microbiome can promote better wound healing and reduce infection risks. Furthermore, research suggests that understanding and potentially modifying the microbiome at the site of transplantation may enhance graft survival and overall skin health. The study of the skin microbiome's role in transplantation is still evolving; at the same time, it puts new horizons at sight in the field of microbiome‐focused therapies in transplantations. Building on this concept, a study conducted by Myles et al. focuses on the role of skin transplantation in AD. In this experimental research, the team transplanted human skin microbiota into murine models to study its influence on disease development. The findings suggest that altering the skin's microbial community can affect inflammation and disease outcome, indicating a potential therapeutic avenue for treating AD through microbiota manipulation. This study contributes to the growing understanding of skin transplantation and microbiome's role in immune responses and inflammatory diseases [60]. It suggests an approach that could lead to the development of microbiota‐based therapies targeted at the underlying microbial imbalances, offering a personalized treatment option for patients suffering from various skin conditions.

### 3.6 Regulatory Aspects

Probiotics and microbial products used in skin care and health applications are regulated by a comprehensive framework developed majorly within the European Union (EU) and the USA, ensuring consumer safety, efficacy, and truthful marketing on a global scale. Products are classified into distinct regulatory pathways determined by their intended use and associated health claims. These pathways may involve authorization at the EU level or notification by the US member states. The classification process is carried out under the supervision of the European Food Safety Authority and the European Medicines Agency. The jurisdiction in the USA where product classification and pre‐market approval are regulated is the Food and Drug Administration. These frameworks exemplify cohesive and rigorous strategies for regulating microbial products and probiotics, with a focus on ensuring consumer safety, utilizing scientific evidence to support health claims, and adhering to ethical marketing standards [61, 62]. Within these frameworks, skincare products fall into various regulatory categories based on the type of the claim that are associated with them and whether they are administrated systemically or topically [63].

### 3.7 Future Directions

The integration of predictive models and machine learning techniques significantly enhances our understanding of the complex relationships within the skin microbiome. These tools can meticulously analyze extensive data from longitudinal studies, pinpointing how variables like diet, lifestyle, and cosmetic consumption interact with the microbiome to influence skin aging [64, 65].

Future research on microbiome‐mediated cosmetic responses covers several key areas. First, longitudinal studies are needed to understand the temporal dynamics of microbiome changes after different cosmetic interventions to better understand microbial resilience and recovery. In the future, integrating biosensors with the skin microbiota will transform healthcare. The advanced sensors will monitor biomarkers for disease detection and analyze interactions within the skin microbiome, offering personalized insights into skin health and wellness [66]. They can also reveal the microbial community's structure, function, and metabolic activity before and after procedures. Studying microbial biomarkers predictive of treatment outcomes and complications will enable personalized treatment strategies and risk assessment for cosmetic surgery patients. Utilizing this technology will enable individuals to actively oversee their well‐being with unprecedented precision and understanding [67, 68, 69]. Additionally, the development and use of 3D skin models can offer invaluable insights into how cosmetic treatments affect the skin microbiome in a controlled, replicable environment, enhancing our understanding of microbial interactions with skin tissues [11, 70]. Moreover, studying the skin microbiome and host immune responses post‐treatment can reveal microbiome‐mediated effects on treatment efficacy and adverse events [4, 23]. Finally, studies on microbiome‐targeted interventions like probiotics or microbiome‐modulating skincare products may improve cosmetic dermatology treatment outcomes and reduce post‐procedural complications. In the light of the infinite potentials that the field of dermatology holds with regard to skin microbiota, future research should focus on the complex relationship between the skin microbiome and cosmetic procedures to develop personalized skin health and aesthetics treatments.

## 4 Conclusion

The correlation between changes in the skin microbiota and the aging process is a significant area of focus in dermatological research, reflecting the complex interplay between microbial shifts and skin health over time. Evidence suggests that aging can lead to alterations in the skin environment, such as reduced lipid production and compromised barrier function, which in turn may favor the growth of certain microbial species while inhibiting others. These changes in the microbial landscape can influence the overall health and appearance of the skin, potentially exacerbating signs of aging. Conversely, emerging research also supports the idea that modifications in the skin microbiota itself might contribute directly to the aging process. For instance, an imbalance in microbial diversity or the overgrowth of pathogenic bacteria can provoke inflammatory responses, lead to oxidative stress, and cause tissue damage, all of which are key factors in the aging of skin. This suggests a potentially cyclical relationship where not only does aging affect microbial composition, but also changes in the microbiota may accelerate aging‐related skin deterioration. However, several limitations in our review must be acknowledged. The observational nature of the reviewed studies prevents a definitive establishment of causality between the skin microbiota and aging. Additionally, the variability in study designs, populations, and methodologies introduces a degree of uncertainty regarding the generalizability of the findings. Finally, the dynamic nature of the skin microbiota, influenced by a wide range of external and internal factors such as diet, lifestyle, and cosmetic use, adds further complexity to isolating the specific effects of microbiota changes on skin aging. These factors must be meticulously addressed in future studies to provide more holistic insights on developing aging skin treatment strategies. However, the current study builds a bulk of literature review that can contribute to future perspectives in this field.

## Author Contributions

Diala Haykal wrote the manuscript, and Hugues Cartier and Brigitte Dréno reviewed it.

## Ethics Statement

The authors have nothing to report.

## Conflicts of Interest

The authors declare no conflicts of interest.

## Data Availability

All information is included in the manuscript.
